# Measuring the Outcome of Biomedical Research: A Systematic Literature Review

**DOI:** 10.1371/journal.pone.0122239

**Published:** 2015-04-02

**Authors:** Frédérique Thonon, Rym Boulkedid, Tristan Delory, Sophie Rousseau, Mahasti Saghatchian, Wim van Harten, Claire O’Neill, Corinne Alberti

**Affiliations:** 1 European and International Affairs Unit, Gustave Roussy, Villejuif, France; 2 AP-HP, Hôpital Robert Debré, Unité d’épidémiologie clinique, Paris, France; 3 Université Paris Diderot, Sorbonne Paris Cité, UMR-S 1123 and CIC-EC 1426, ECEVE, Paris, France; 4 INSERM, U 1123 and CIC-EC 1426, ECEVE, Paris, France; 5 AP-HP, Hôpital Bichat, Département d’Epidémiologie et de recherche clinique, Paris, France; 6 Direction de la Recherche Clinique, Gustave Roussy, Villejuif, France; 7 Centre Hygée, Department of Public Health, Lucien Neuwirth Cancer Institute, CIC-EC 3 Inserm, IFR 143, Saint-Etienne, France; 8 The Netherlands Cancer Institute, Amsterdam, the Netherlands; Stanford University, UNITED STATES

## Abstract

**Background:**

There is an increasing need to evaluate the production and impact of medical research produced by institutions. Many indicators exist, yet we do not have enough information about their relevance. The objective of this systematic review was (1) to identify all the indicators that could be used to measure the output and outcome of medical research carried out in institutions and (2) enlist their methodology, use, positive and negative points.

**Methodology:**

We have searched 3 databases (Pubmed, Scopus, Web of Science) using the following keywords: [Research outcome* OR research output* OR bibliometric* OR scientometric* OR scientific production] AND [indicator* OR index* OR evaluation OR metrics]. We included articles presenting, discussing or evaluating indicators measuring the scientific production of an institution. The search was conducted by two independent authors using a standardised data extraction form. For each indicator we extracted its definition, calculation, its rationale and its positive and negative points. In order to reduce bias, data extraction and analysis was performed by two independent authors.

**Findings:**

We included 76 articles. A total of 57 indicators were identified. We have classified those indicators into 6 categories: 9 indicators of research activity, 24 indicators of scientific production and impact, 5 indicators of collaboration, 7 indicators of industrial production, 4 indicators of dissemination, 8 indicators of health service impact. The most widely discussed and described is the h-index with 31 articles discussing it.

**Discussion:**

The majority of indicators found are bibliometric indicators of scientific production and impact. Several indicators have been developed to improve the h-index. This indicator has also inspired the creation of two indicators to measure industrial production and collaboration. Several articles propose indicators measuring research impact without detailing a methodology for calculating them. Many bibliometric indicators identified have been created but have not been used or further discussed.

## Introduction

There is an increasing demand for research evaluation. Research funders want to assess whether the research that they fund has an impact [1]. In addition to demonstrate accountability and good research governance, research funding organizations need to build an evidence base to inform strategic decisions on how to fund research [2]. According the Canadian Academy of Health Sciences, evaluation of research is carried out for three main purposes: accountability purposes, advocacy purposes, and learning purposes. Evaluation for accountability is usually performed by funders to assess whether the outcome of their funding has fulfilled its anticipated aim and has strong links to value-for-money issues. Evaluation for advocacy aims to increase awareness of the achievements of a research organisation in order to encourage future support. Evaluation for learning is an inward looking process that aims to identify where opportunities, challenges and successes arise for the research performed in an institution [3].

Some of the existing evaluation systems include assessments by national agencies, the Organisation for Economic Cooperation and Development (OECD) Frascati Manual, the UK Research Assessment Exercise, the Shanghai ranking, etc… Those systems use a set of different indicators, sometimes complemented by peer-review. An indicator is defined as ‘a proxy measure that indicates the condition or performance of a system’ [4]. Indicators are said to be more objective than peer-review assessment [5].

Nevertheless, there is an increasing call for evaluation of medical research in terms of its benefits to patients [6–12]. From a policy making perspective, this vision particularly applies to health care institutions, such as university hospitals or comprehensive cancer centers where research and patient care are integrated and sometimes carried out by the same professionals. In the view of designing an evaluation system to measure the outputs, outcomes and impact of medical research, it is necessary to have first an overview of all possible indicators, as well as their positive and negative points.

Some reviews of indicators measuring research production have been conducted [13–16]. Those review focus mainly or exclusively on bibliometric indicators or research input indicators (such as research funding). The scope of our systematic review is different as it focuses exclusively on indicators measuring the production, output and outcome of medical research, and that it intends to go beyond bibliometric indicators in order to include indicators measuring long-term impact of medical research. In this article we define outputs as “the immediate tangible results of an activity” and outcomes as “longer-term effects such as impact on health” [17]. We use the definition of impact proposed by the Canadian Institute of Health Research: “In the context of evaluating health research, the overall results of all the effects of a body of research have on society. Impact includes outputs and outcomes, and may also include additional contributions to the health sector or to society. Impact includes effects that may not have been part of the research objectives, such as contributions to a knowledge based society or to economic growth” [18]. In this article we make a distinction between scientific impact and health service impact.

We conducted a systematic review with the following objectives: (1) to identify all existing indicators that can be used to measure the output or outcome of medical research performed by institutions, and (2) to list, for all indicators, their positive and negative points, as well as the comments on their validity, feasibility and possible use.

## Methodology

We wrote a protocol prior to the start of the study. We chose to undertake a review of all indicators, including those used to measure research areas outside the biomedical field.

### 1. Search strategy

We searched in Pubmed, Scopus and Web of science, using the following terms: [“research outcome*” OR “research output*” OR “research impact*” OR bibliometric* OR scientometric* OR “scientific production*”] AND [indicator* OR index* OR evaluation* OR metric* OR “outcome* assessment”], as terms in the abstract, title or keywords, with no time limit. On those three databases we applied filters on language (including only articles written in French or English) and on type of document (including only articles and reviews).

Through snowballing, we added articles that seemed relevant in the bibliography of selected articles. Two of us (FT and RB) undertook the search independently, assessed articles on the basis of title and abstract and compared our search results. Differences in results were discussed and resolved.

### 2. Inclusion criteria

Considering the scope of our project to develop indicators designed to measure the production and outcome of research undertaken by institutions (such as hospitals, research centres or research units…) we set up inclusion and exclusion criteria accordingly. We included articles written in French or English, that presented, discussed or evaluated indicators measuring the scientific production of an institution. We excluded:

- articles that presented or assessed only indicators measuring research inputs (such as funding or human resources),
- articles that presented, discussed or evaluated indicators measuring the scientific production of an individual researcher or a country,
- articles that presented a bibliometric or scientometric study,
- articles that presented, discussed or evaluated indicators measuring the quality of a scientific journal and
- articles in languages other than French or English.

We assessed the relevance or quality of articles using a list of criteria. Each article should at least contain one criterion to be selected:

- The article presents an indicator and clearly describes how it is calculated
- The articles evaluates the validity or reliability of the indicator
- The articles evaluates the feasibility of the indicator
- The article discusses the validity of the indicator to measure scientific production
- The article contains proof on the implementation: It describes the possible perverse consequences of measuring the indicator
- The article relates how the indicator was developed and implemented

We noted the reason for exclusion of articles and presented the results according to the PRISMA reporting method [19].

### 3. Information retrieved

We developed and tested two data extraction forms to collect and organise data about (1) the type of articles selected and the information they produced and (2) details of indicators presented in the articles. The templates of the data extraction forms are available in annex 1 and annex 2 (S1 Appendix; S2 Appendix).

Using the first form, we retrieved information about each article: the article presents the results of surveys to select one/several indicator(s) (Yes/No), the article relates the development of one/several indicator(s) (Yes/No), the article evaluates the feasibility of one/several indicator(s) (Yes/No), the article evaluates the validity of one/several indicator(s) (Yes/No), the article evaluates the impact of measuring one/several indicator(s) (Yes/No), the article presents any other form of evaluation of the indicator (Yes/No), number and names of indicators. We also collected for each article the name of the journal in which it was published, the impact factor and type of journal, and the domain of research.

Using the second data extraction form, we retrieved the following information for each article: name of the indicator, references in articles, definition of the indicator and details how it is calculated, rationale of the indicator (why it was created), in what context the indicator is used, positive and negative points of the indicator, impact or consequences of measuring the indicator and any additional comments.

When the article presented a mix of relevant indicators and irrelevant indicators (example: inputs indicators), we only retrieved information about the relevant indicators.

The full text of every article was read and data were extracted a first time by FT and proof-read by different authors. The allocation of articles to proofreaders was executed randomly, however the number of articles read differed by reviewers: TD reviewed 16% articles (N = 12), SR reviewed 48% of articles (N = 36), RB reviewed 18% articles (N = 14), CO reviewed 18% articles (N = 14). All differences in opinion were discussed and resolved through discussion.

## Results

### 1. Number and characteristics of articles

**Fig 1:**
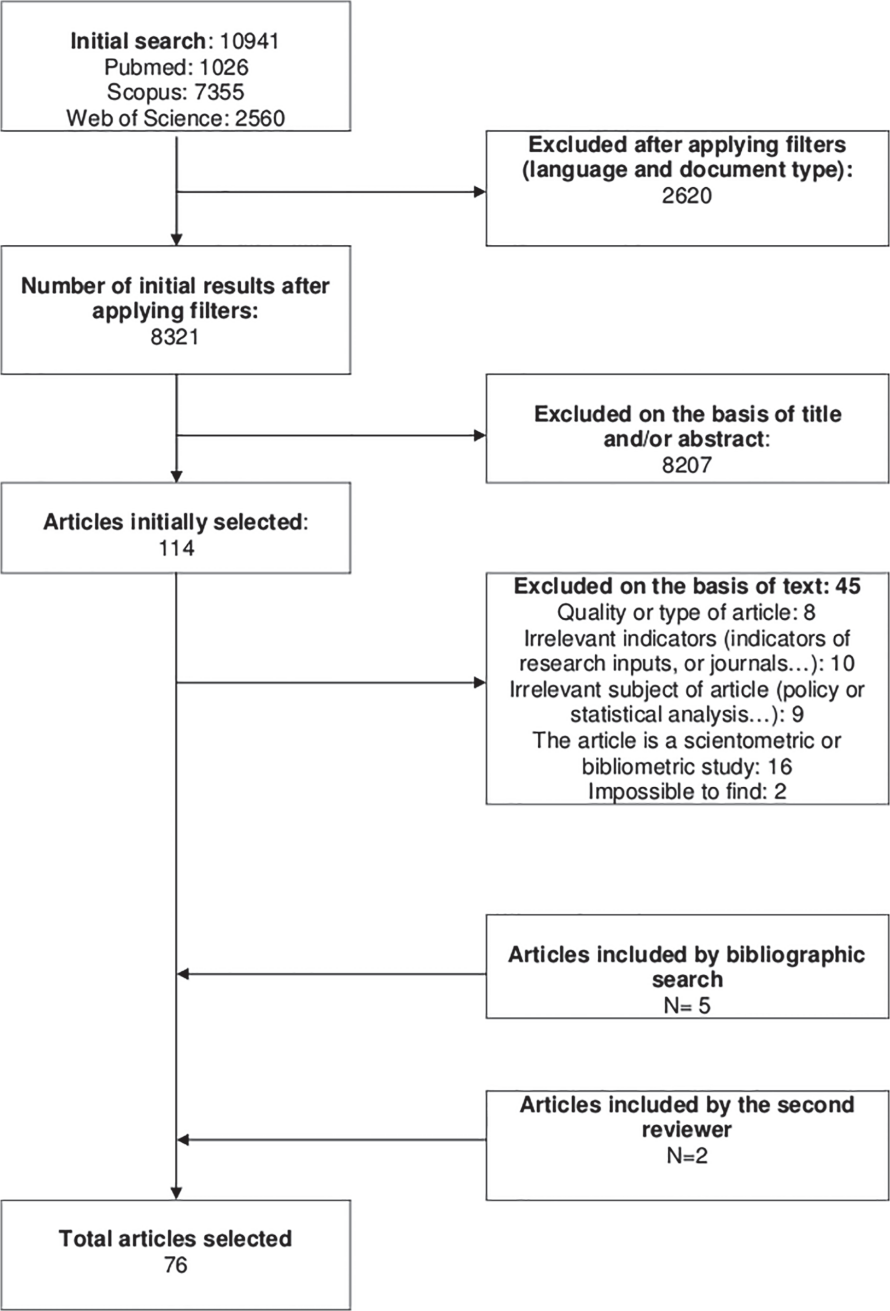
PRISMA flowchart.

After applying filters we retrieved 8321 articles and selected 114 on the basis of title and abstract. Then, 45 articles were excluded after reading the full text, either because of the quality or type of the article (such as commentaries), or because the indicators presented were irrelevant (such as indicators of research inputs, indicators of a journal…), because the articles presented a scientometric or bibliometric study and did not contain indicator description, or because the subjects of the articles were irrelevant (articles presenting a policy analysis on research evaluation or exclusively relating the development of indicators). In addition, 5 articles were added by reference, 2 articles were added by the second reviewer. A total of 76 articles were selected. Fig 1 describes the selection of articles (Fig 1).

The articles were found in 42 different journals and the median impact factor of all articles was 2.1. Almost half of the articles (N = 35) emanated from journals specialized in information science or scientometrics. Overall, 36% of other articles (N = 28) belonged to a medical or public health journal. Table 1 shows the characteristics of the journals in which the articles were published, as well as the research area covered by the article (Table 1: Type of journals and area of research measured by indicators).

**Table 1:** Type of journals and area of research measured by indicators.

Characteristics (N = 76)	N (%)
**Type of journal**	
Information science/scientometrics	35 (46%)
Medical speciality	16 (21%)
Public health	6 (8%)
General science	5 (7%)
General medicine	6 (8%)
Biology	4 (5%)
Others (social science, research methodology, pharmacological)	4 (5%)
**Research area measured by indicator(s)**	
General	47 (62%)
Biomedical research	16 (21%)
Public health	3 (4%)
Psychiatry	2 (3%)
Surgery	2 (3%)
Cancer research	1 (1%)
Chemistry	1 (1%)
Biobanks	1 (1%)
Bioinformatics	1 (1%)
Technological transfer	1 (1%)
Translational research	1 (1%)

### 2. Content of selected articles

Among all the articles found, 1 article presented the results of a survey to select indicators, 5 articles related the development of one or more indicator(s), 12 evaluated the feasibility of one or more indicator(s), 24 evaluated the reliability or validity of one or more indicator(s), no studies evaluated the impact of measuring one or more indicator(s). Among all articles, 34 studies undertook any other form of evaluation of one or more indicator(s).

### 3. Indicators

We found 57 indicators presented or discussed in all the articles. We classified those indicators into 6 categories: indicators of research activity, indicators of scientific production and impact, indicators of collaboration, indicators of dissemination, indicators of industrial production and indicators of health service impact, (Table 2: Number of indicators by category). Table 3 summarises the indicators identified, the number of articles in which those indicators are discussed, whether a definition and a methodology for indicator measurement are provided, and whether the positive and negative points of this indicator are mentioned (Table 3: Summary of indicators identified). Complete synthesis of the indicators is enlisted in annex 3. This synthesis is based on reported data and includes the definition of each indicator, the rationale for its creation or its use, and its positive and negative points (S3 Appendix).

**Table 2:** Number of indicators by category.

**Category of indicator**	**Number of indicators presented (n = 57)**
Indicators of activity	8 (14%)
Indicators of scientific production and impact	24 (42%)
Indicators of collaboration	5 (9%)
Indicators of dissemination	4 (7%)
Indicators of industrial production	7 (12%)
Indicators of health service impact	9 (16%)

**Table 3:** Summary of indicators identified.

**Category**	**Indicators**	**Number of articles**	**Rationale provided**	**Definition provided**	**Methodology or calculation provided**	**Positive and negative points discussed**
**Indicators of research activity**	Number of clinical trials	2	Yes	Yes	No	No
	Number of patients in clinical trial	1	No	No	No	No
	Number of biological samples transmitted	1	No	No	No	No
	Number of research projects ongoing	1	No	No	No	No
	Number of biomarker identified	1	No	No	No	No
	Number of assays developed	1	No	No	No	No
	Number of databases generated	1	No	No	No	No
	Number of visits to the EXPASY server	1	Yes	Yes	Yes	Yes (+/-)
**Indicators of scientific production and impact**	h-index	31	Yes	Yes	Yes	Yes (+/-)
	Number of publications	16	Yes	Yes	Yes	Yes (+/-)
	Number of citations	14	Yes	Yes	Yes	Yes (+/-)
	Journal impact factor	10	Yes	Yes	Yes	Yes (+/-)
	g-index	6	Yes	Yes	Yes	Yes (+/-)
	Crown indicator	4	Yes	Yes	Yes	Yes (+/-)
	m-quotient	4	Yes	Yes	Yes	Yes (+/-)
	hg-index	3	Yes	Yes	Yes	Yes (+)
	Citer h-index (Ch-index)	2	No	Yes	Yes	Yes (+)
	Mean citations per papers	2	No	Yes	Yes	Yes (+/-)
	b-index	2	No	Yes	Yes	No
	AWCR (age-weighted citation ratio)	1	Yes	Yes	Yes	Yes (+/-)
	Mean normalised citation score	1	No	Yes	Yes	No
	z-factor	1	Yes	Yes	Yes	Yes (+)
	j-index	1	Yes	Yes	Yes	Yes (+/-)
	SP-index	1	Yes	Yes	Yes	Yes (+)
	Number of publications in the top-ranked journals	1	Yes	Yes	Yes	Yes (+/-)
	x-index	1	Yes	Yes	Yes	Yes (+/-)
	Central index	1	Yes	Yes	Yes	Yes (+/-)
	w-index	1	Yes	Yes	Yes	Yes (+/-)
	e-index	1	Yes	Yes	Yes	No
	r-index	1	Yes	Yes	Yes	Yes (+)
	m-index	1	Yes	Yes	Yes	Yes (+)
	Q^2^ index	1	Yes	Yes	Yes	No
**Indicators of collaboration**	Number of co-authored publication	4	No	Yes	Yes	No
	Number of articles with international collaboration	2	No	Yes	Yes	No
	Proportion of long-distance collaborative publication	1	No	Yes	Yes	No
	Partnership Ability Index (PHI-index)	1	No	Yes	Yes	Yes (+)
	d-index (dependence degree)	1	Yes	Yes	Yes	Yes (-)
**Indicators of dissemination**	Reporting of research in the news/media	4	Yes	Yes	No	Yes (+/-)
	Citation in medical education books	2	Yes	Yes	No	Yes (+/-)
	Number of presentations at key selected conference	2	Yes	Yes	No	No
	Number of conference held	1	No	No	No	No
**Indicators of industrial production**	Number of patents	6	Yes	Yes	Yes	Yes (+/-)
	Number of public-private partnerships	2	Yes	Yes	No	No
	Patent citation count	2	Yes	Yes	Yes	Yes (+/-)
	Number of spin-off companies created	2	Yes	Yes	No	Yes (-)
	Citation of research in patents	2	Yes	Yes	No	No
	Number of papers co-authored with the industry	1	Yes	Yes	Yes	Yes (+/-)
	Patent h-index	1	Yes	Yes	Yes	Yes (+/-)
**Indicators of health service impact**	Citation of research in clinical guidelines	7	Yes	Yes	suggested	Yes (+/-)
	Contribution to reports informing policy makers	4	Yes	No	No	No
	Citation of research in policy guidelines	3	Yes	Yes	No	Yes (+/-)
	Patients outcomes	3	Yes	Yes	No	Yes (-)
	Public knowledge about a health issue	2	No	Yes	No	Yes (-)
	Changes in legislation/regulations	2	Yes	Yes	No	Yes (+/-)
	Generation of clinical guidelines	1	Yes	Yes	No	No
	Changes in clinical practice	1	Yes	Yes	suggested	Yes (+/-)
	Measures of improved health services	1	Yes	Yes	No	No

In the ‘positive and negative points discussed’ column: Yes(+) = Yes, only positive points are mentioned, Yes(-) = Yes, only negative points are mentioned, Yes (+/-) = Yes, positive and negative points are mentioned

#### • Research activity indicators

We found 8 indicators measuring research activity. Indicators of research activity describe the size and diversity of the research pipeline and assess progress towards established milestones. With the exception of one indicator, most of those indicators are presented in one article [20] that does not discuss their positive and negative points. A general comment warns against using solely those kinds of indicators as they would reward an organisation that keeps projects in the pipeline even if they don’t appear to be destined for a successful outcome.

#### • Indicators of scientific production or impact

Most of the indicators we found are indicators of scientific production and impact (N = 23). Definition and methodology were provided for all those indicators. For most of them, rationale (N = 20), positive points (N = 20), and negative points (N = 14) were mentioned. The most discussed indicator of that category is the h-index (31 articles). This indicator was created by Hirsch in 2005 and combines measures of quantity (number of publications) and impact (number of citations). It was created to overcome the flaws of other classical bibliometric indicators such as the number of publications (which does not measure the importance of papers), the number of citations (which may be influenced by a small number of very highly cited papers), or the number of citations per paper (which rewards low productivity) [21]. Some of the reported advantages of the h-index include its easy calculation [22–24], its insensitivity to a few highly or infrequently cited papers [22], and the fact that it favours scientists that publish a continuous stream of papers with good impact [25]. Studies have tested this indicator and found evidence that it can predict the future achievement of a scientist [26]; it correlates with peer review judgement [27] and shows a better validity than publication count or citation count alone [28]. Some of its reported flaws include its failure to take into account the individual contribution of each researcher [29], or its low resolution (meaning that several researchers can have the same h-index) [30]. As a result, several indicators have been created to overcome those flaws: the j-index [23], the central index [31], the w-index [32], the e-index [30], the r-index [33], the m-index [34], the m-quotient [24], the citer h-index [35], the q^2^ index [34], the g-index [24] and the hg-index [36].

One criticism of several indicators based on citations (such as the h-index, number of citations, journal impact factor…) is that citation practices vary between disciplines [5]. In the field of medicine, for example, basic research is cited more than clinical research [37]. Hence the creation of indicators adjusting the citation rate by disciplines such as the mean normalised citation score [38], b index [24] and the crown indicator [13].

Another indicator subject to much controversy is the journal impact factor. Although this indicator was created to measure the visibility of a journal, it is commonly used to measure scientists and institutions. Some of its criticisms point out that it is influenced by discipline, language, open access policy [5], and can be manipulated with the number of articles [39]. It has been suggested this indicator should be used only to measure journals and not scientists.

#### • Indicators of collaboration

Five indicators were found to measure research collaboration: the dependence degree, the partnership ability index, the number of co-authored publications, the number of articles with international collaboration and the proportion of long-distance collaboration. The rationale for the use of those indicators is that research benefits from collaboration between institutions because it brings new ideas and methods and multifaceted expertise can be reached [40], and therefore evaluation metrics should focus on the interactions between researchers rather than on the outputs of individual researchers [41]. A definition and methodology was provided for all those indicators, but we found little critical discussion about the advantages and disadvantages of using them.

#### • Indicators of dissemination

There were 4 indicators measuring the dissemination of research: citation in medical education books, number of presentations at key selected conferences, number of conferences organised and reporting of research in news or media. Although the rationale and definition was provided for 3 of them, none proposed a methodology. The most discussed indicator was the reporting of research in the news/media. The rationale for the development or use of that indicator is that media are often influential in terms of public opinion and public formation and media reporting of research allows patients to be better informed [7]. It is argued that scientists interacting most with mass media tend to be scientifically productive and have leadership roles and media have a significant impact on public debate [37]. However, criticisms of this indicator include its bias (for example, clinical research is over-represented), its lack of accuracy [37] and lack of evidence that it leads to an actual policy debate [9].

#### • Indicators of industrial production

There were 7 indicators measuring industrial production: the number of public-private partnerships, the number of patents, the number of co-authored publications, the number of papers co-authored with the industry, the patent citation count, the patent h-index, the citation of research into patents, and the number of spin-off companies created. The most widely discussed indicator was the number of patents with 6 articles discussing it and 3 indicators derived from it (patent citation count, patent h-index and citation of research in patents). Although it is mentioned that patent protection enhances the value of an organisation’s output and attracts future commercial investment, more criticisms of this indicator are acknowledged, such as its lack of reliability in measuring patent quality and subsequent impact, and its adverse effects on the quality of patents produced by a university [9]. To overcome this flaw, the patent citation count is sometimes used; however most patents are never cited or cited only once or twice. As a corrective, the patent h-index has been created, that combines measures of quantity and impact of a patent. Other measures of industrial production or collaboration are scarcely discussed and rarely used.

#### • Indicators of health service impact

We found 9 indicators measuring research impact on health or health service. Several articles proposed to measure the impact of medical research in terms of various measures of patients’ outcomes (such as mortality, morbidity, quality of life…). However those indicators are very challenging to measure [12], and pose the problem of attribution (how to link health improvements to one particular research finding) [9]. Other intermediate outcome indicators for health research have been suggested, such as changes in clinical practice, improvement of health services, public knowledge on a health issue, changes in legislation and clinicians’ awareness of research. However no article gave a clear methodology for calculating those indicators, or a way to tackle the attribution problem. The authorship of clinical guidelines or researchers’ contribution to reports informing policy makers are other possible indicators of medical research outcome, although there is little discussion on their advantages and disadvantages. More has been written on two indicators: citation of research in clinical guidelines and citation of research in policy or public health guidelines. The indicator ‘citation of research in clinical guidelines’ has been most widely discussed in that category. It is reported as being easy to calculate and correlated with other quality indicators such as impact factor [37]. But this indicator can only measure research several years after it has been published. It also favours clinical research compared with basic research.

## Discussion

### Interpretation of results

The aim of our study was to obtain a comprehensive view of existing indicators used to measure the output or outcome of medical research. We found 57 indicators developed, with a majority of bibliometric indicators present in most articles. This finding is consistent with previous review of research indicators [16]. We also found a diversity of indicators, measuring different elements of medical research. We decided to classify them into 6 categories (indicators of research activity, indicators of scientific production, indicators of collaboration, indicators of dissemination, indicators of industrial production and indicators of health research impact). Few articles discussed research activity indicators. The positive and negative points of those indicators were not discussed and no methodology for calculating the indicator was provided, except for the indicator ‘number of visits to EXPASY server’. Therefore the second objective of our study (noting the positive and negative points and remarks about feasibility, validity or use of indicators) could not be completed for this category of indicators. More research is needed on this aspect.

Not surprisingly, bibliometric indicators of scientific production were the most represented and discussed category of indicators. The most discussed indicator of that category was the h-index. Several indicators have been developed to improve it or complement it. The h-index has inspired the creation of different indicators belonging to other categories, such as the patent h-index or the partnership ability index. However the focus of bibliometric to measure research production has been criticized. Indeed some of the critics point out that those indicators do not reflect truly the impact of a research work on the scientific community and on public health. For example, Edward Lewis, a scientist famous for his work on the role of radiation on cancer and who won a Nobel Prize had a small publication count and a very low h-index [42]. Another one is the discovery of the pathogenic role of the *Helicobacter Pylori* bacterium, which was first published in the *Medical Journal of Australia*, a journal with an impact factor below 2 [43].

Five indicators measure inter-institutional research collaboration. Some of those indicators are used alongside other bibliometric indicators in evaluation systems such as the Leiden Ranking [38]. According to Abramo [44], there has been a trend towards an increase in collaboration between institutions. This trend could be attributed to many factors, such as specific policies favouring research collaboration, specifically the EU Research Framework Programmes at the international level [44], or the increased division of labour among scientists [45]. Several studies have measured the impact of co-authored papers and found that they are more highly cited than papers authored by a single institution, and papers with international co-authorships have an even higher citation rate [44–48]. However, it has also been argued that co-authorship is a poor indicator of actual collaboration [49; 50].

In the category ‘indicators of dissemination’, one indicator has been widely reported upon is the citation of research in the mass media. The criticism on its bias and lack of accuracy is consistent with other researches. A study on the reporting of cancer research on BBC website [51] has shown that this media does not broadcast themes of cancer research consistently with their epidemiological burden. For example many cancers such as lung cancer and upper gastro-intestinal tract fare poorly in their media exposure despite an important incidence or mortality. Another research on the reporting of mental health research found similar results [52]. And a study found evidence of poor media reporting of health interventions despite recent improvements [53]. We found some indicators of health service impact. But they seem difficult to measure and present the challenge of attributing particular research findings to health improvements.

### Strength and limitations of the study

This study has limitations. We have been able to identify indicators belonging to a broad spectrum and that can measure the outcome of medical research from various perspectives. However this is also one limitation of the study. We chose to design our analysis in order to obtain a broad view of indicators. However, we might not have been able to give an in-depth analysis of the bibliometric indicators. They were not the focus of our study.

We have decided to classify the indicators found into 6 categories but several indicators could belong to more than one category.

### Policy implications

Several lessons can be drawn from this study. Given the fact that all indicators have flaws or are incomplete, several studies [54; 55; 27] stressed the importance of using a mix of several indicators rather than just one to measure research outputs. An evaluation system should follow this recommendation. Another important step in the development of indicators is to assess their validity and feasibility. According to the OECD, an indicator is valid when it accurately measures what it is intended to measure and it is reliable when it provides stable results across various populations and circumstances [56]. There are three conditions to assess the feasibility of an indicator: existence of prototypes (whether the measure is in use), availability of internationally-comparable data across countries and cost or burden of measurement [56].

## Conclusion

We have drawn a comprehensive list of indicators measuring the output and outcomes of medical research. Not all indicators are suitable to evaluate the outcome of translational research carried out in health facilities. In order to operate a selection of indicators, we plan on investigating the view of concerned researchers about the key indicators to select. We also need to test the feasibility and validity of the selected indicators.

## Supporting Information

S1 PRISMA Checklist PRISMA checklist. (DOC)

S1 Appendix Data Extraction Form 1. (DOC)

S2 Appendix Data Extraction Form 2. (DOC)

S3 Appendix Details on each indicator. (DOC)

S1 Protocol Study protocol. (DOC)
