# Supplementary material for: Measuring the Outcome of Biomedical Research: A Systematic Literature Review
Source: PLoS One. 2015 Apr 2;10(4):e0122239. doi: 10.1371/journal.pone.0122239 (PMC4383328; doi:10.1371/journal.pone.0122239)
Supplement: S1 Appendix — (DOC) [file pone.0122239.s002.doc]

**Annex 1: Data extraction form for each article:**

| Full reference of the article |  | | |
| --- | --- | --- | --- |
| Is the article : (Yes/No) | | | |
| Presenting the results of surveys to select indicators (such as Delphi survey) | | |  |
| Relating the development of an indicator | | |  |
| A study on the feasibility of an indicator | | |  |
| A study on the validity or reliability of an indicator | | |  |
| An evaluation of the impact of developing or collecting an indicator | | |  |
| Any other form of evaluation of an indicator | | |  |
| Number of indicators presented in this article: | | |  |
| Name of this (or those) indicator(s): | |  | |
