# Supplementary material for: Measuring the Outcome of Biomedical Research: A Systematic Literature Review
Source: PLoS One. 2015 Apr 2;10(4):e0122239. doi: 10.1371/journal.pone.0122239 (PMC4383328; doi:10.1371/journal.pone.0122239)
Supplement: S2 Appendix — (DOC) [file pone.0122239.s003.doc]

**Annex 2: Data extraction form for each indicator:**

| **Name of the indicator** |  |
| --- | --- |
| Reference of the article(s) discussing that indicator |  |
| Definition and calculation of indicator |  |
| Rationale of indicator (why was it created) |  |
| How the indicator is used |  |
| Positive points of the indicator |  |
| Negative points of the indicator |  |
| Impact of using or measuring the indicator |  |
| Other comments |  |
