# Supplementary material for: Measuring the Outcome of Biomedical Research: A Systematic Literature Review
Source: PLoS One. 2015 Apr 2;10(4):e0122239. doi: 10.1371/journal.pone.0122239 (PMC4383328; doi:10.1371/journal.pone.0122239)
Supplement: S3 Appendix — (DOC) [file pone.0122239.s004.doc]

**Annex 3: Analysis of each indicator identified**

1 : Indicators of activity

| **Name of the indicator** | **References** | **Definition of indicator** | **Rationale and/or use of this indicator** | **Positive points** | **Negative points** |
| --- | --- | --- | --- | --- | --- |
| Number of patients in clinical trial | [52] | Number of patients included in a clinical trial | This is one of the indicator of creation that should describe the size and diversity of the research pipeline and assess progress towards established milestones | - | An organization can keep projects going even if they do not appear successful for a positive outcome |
| Number of clinical trials | [52; 48] | - | Clinical trials are a key research activity with the potential to improve human health | - | - |
| Number of biological samples collected | [52] | - | - | - | - |
| Number of biological samples transmitted | [52] | - | - | - | - |
| Number of research projects ongoing | [52] | - | - | - | - |
| Number of biomarker identified | [52] | - | - | - | - |
| Number of assays developed | [52] | - | - | - | - |
| Number of databases generated | [52] | - | - | - | - |
| Number of visits to the EXPASY server | [31] | The EXPASY (Expert Protein Analysis System) is a virtual research infrastructure for bioinformatics. This indicator measures the number of visits to this platform | - It is interesting to have more direct indicators of research activity - Biological databases are important to advance | It is correlated with the number of publications so is a good proxy for research productivity | Calculates only one aspect of research activity |

**2: Indicators of scientific production and impact**

| **Name of the indicator** | **References** | **Definition of indicator** | **Rationale and/or use of this indicator** | **Positive points** | **Negative points** |
| --- | --- | --- | --- | --- | --- |
| Number of publications | [5;15; 17; 26; 27; 28; 35; 40; 46; 52; 57;67; 73; 55; 34; 22] | Number of peer-reviewed publications authored by the institution/ researcher in one definite period of time | Published results of research play a fundamental role in the circulation and exploitation of knowledge | - easy to measure - it offers background for other evaluations - measures productivity - There are few studies that show a correlation between the number of publications and other research performance measures | - it only measures quantity, not quality - it does not measure impact or importance of papers |
| Number of citations | [1;15; 26;27;28; 35; 40;42;52;67;71; 55; 34; 22] | Counting of the number of citations received by a researcher or group of researchers from published articles within a time span | Assumption that articles of good quality are cited more often.  Measures the contribution of a piece of work on the advancement of knowledge. | - It correlated positively with other indicators of performance, such as number of publications, peer-review, research funding and H-index - Measures total impact - Can predict future achievement of scientists - Can be adjusted to improve its accuracy | - it is argued that this indicator is an indicator of market share and not quality - can be affected by negative citations or self-citations - hard to find - may be inflated by a small number of ‘big hits’ which may not represent the author, especially if he is a co-author - varies between disciplines - citations are good for basic research but underestimate clinical research - It can increase the practice of self-citation |
| h-index | [2; 4; 8; 9; 11;12;15; 16; 19; 23; 24; 26; 27;28;30; 32; 33; 36; 41; 44; 49; 50; 53; 60; 63; 64; 68; 75; 55; 34; 22] | the number of papers with citation number ≥ h as a useful index to characterise the scientific output of a researcher | This index combines quantitative measure (number of papers) and qualitative (number of citations). It is supposed to overcome the disadvantages of other commonly used indicators | - easy to calculate - overcomes the limitations of the previous indicators - measures both quantity and impact - is insensitive to one or several outstandingly cited papers and to infrequently cited articles - favours scientists that publish a continuous stream of papers with good impact - Can predict future achievement of scientists - Correlates with peer-review judgement - studies found that it performs better for research evaluation than publication count or citation count alone - positive correlation between h-index and peer-review rating - is valid for groups or institutions - is valid for healthcare research | - unable to distinguish between active and retired researchers - does not differentiate between disciplines where citation patterns differ - is dependent on the length of existence - is different according to the database used - the individual contribution of each author is not taken into account - is influenced by self-citation - less predictive accuracy than the simpler measure of mean citations per paper - does not differentiate between different types of articles (original research, versus review) - is insensitive to highly cited papers - is sensitive to homonyms conflicts - Low resolution (many scientists have the same h-index) |
| Journal impact factor | [1; 44; 46; 51; 52; 59; 61; 67; 34; 55] | A ratio of number of citations to number of citable items of a journal | This indicator measures the visibility of a journal | - Gives a global view - Simple calculation - It does not reward journals just because they publish a lot - Insight into recent performance - Changes through time visible - Reasonable results - Acceptance | - Is influenced by publication language, document type, citation patterns, open access journals, fast-track publication etc… - Does not capture enough of the multidimensional phenomena of a journal’s influence. - There is confusion and concern over the definition of citable items - The journal impact factor may be inflated in the numerator (editorials or letters, not accounted for in the denominator) - It is misleading concerning central tendency. - Inequalities in journals according to their format (review or multidisciplinary) - Two-year citation window is too short and penalizes some fields - Only for journals indexed in Thomson Reuters - Can be manipulated by the number of articles published - It is meant to measure a journal and should not be used to measure scientists |
| AWCR (age-weighted citation ratio) | [24] | A measure of the average number of citations for an entire body of work adjusted for the age of each individual paper | Citations increase with time | - Actual number of citations are taken into account - Makes use of the age of publication - Can be used with h-index to improve accuracy | Does not apply to items where age has no meaning |
| Mean normalised citation score | [71] | Average number of citations of publications of a university, normalised for differences between scientific fields, between publication years, and document types (article, letters and reviews) | - | - | - |
| Citer h-index (Ch-index) | [19; 20] | The number such that for a general group of papers, ch papers are cited by no more than ch different citers. Similar to the definition of h-index, except that received citations are replaced by different citers | - | - it is not influenced by self-citations - citations do not have the same importance (some papers have a small number of citations that interest a large number of researchers) - it is less influenced by database errors - can be used to evaluate research groups - gives credit to all co-authors of the group - May complement the h-index | - |
| z-factor | [76] | The z-index takes into account both the number of publications and the impact factor of the journals in which they are published | When there is focus solely on the impact factor, problems occur when scientists strive to publish in high impact journals by constantly changing their research topics | - Can be calculated annually - Takes the focus off the simple impact factor | - |
| g-index | [2; 11; 16; 24; 63; 66] | A set of papers has a g-index if g is the highest rank such that top *g* papers have, together, at least g² citations | Meant to be an improvement compared with the h-index because it reduces the influence of the total number of documents in the final index | - It is successful in evaluating the production of a researcher, incorporating the actual citations of his papers - The g-index is more sensitive to assess selective researchers - The weighting of citations is taken into account - The total number of publications is not taken into account - It has a greater discriminating power | - It can be influenced by a very successful paper - Difficulty of collection of all citations and documents of a scientist - Existence of different documents with different impact - Problems of self-citations - Inability of the index to compare scientists of different fields - The g-index should be interpreted in terms of the impact of the researcher’s core papers, For overall publications, the h-index is better |
| j-index | [63] | The j-index is derived from the h-index but also takes into account excess of publications in the h-core and the distribution of citations | Designed to overcome the disadvantages of the h-index | The j-index removes the two main drawbacks of the h-index: (a) it removes the degeneracy and (b) it takes into account the excess of the number of citations. (Degeneracy is when two scientists with a significant difference in the total number of citations in the h-core papers may have the same h-index) |  |
| SP-index | [13] | Index that incorporates number of papers, number of citations and impact factor of publications | The commonly used indicators have flaws | - Easy to calculate |  |
| Number of publications in the top-ranked journals | [15] | Number of publications in highest quality journals according to their impact factor | To overcome the limitations of different citation practices in different disciplines | - Good to compare different research disciplines | Does not address the effect of group size |
| Mean citations per papers | [27; 28] | Number of citations per articles |  | It allows for the comparison of scientists of different ages | - Hard to find - Rewards low productivity and penalises high productivity - Not the best to predict future achievements of a scientist |
| x-index | [48] | Index calculated from the number of national articles in top 1% and 0.1% of highly cited articles | Finding an indicator that correlates with the number of Nobel Prize achievements | - It has a very good correlation with Nobel Prize achievements | Because of its subtraction term it can be negative |
| Central index | [14] | Indicator that corrects biases of mass collaboration and punctual success by not considering all production and impact | Created in response to many critics of the h-index (it depends on the scientific field and the number of collaborations, it correlates with the number of publications, all citations of the most cited article are not considered in this index and the index penalizes selective researchers). This indicator is a complement of the h-index that increases its consistency and favours selective researchers | - It corrects one flaw of the h-index: it penalizes selective researchers | It is a complement of the h-index, not a substitute |
| w-index | [74] | If all the papers of a scientist are ranked in descending  order of the number of citations they received, the w-index is  the highest number of papers one has that have each received  at least 10*w* or more citations | To improve the h-index’s accuracy while keeping its simplicity | - The w-index can better represent the real impact of a scientist’s top papers | Differences in databases can give different results |
| e-index | [75] | An h-index with ignored excess citations | Many indicators have been proposed to overcome the limitations of the h-index but two disadvantages have not been sufficiently overcome: loss of citation information and low resolution (many scientists have the same h-index) |  | - |
| Hg-index | [2; 18; 63] | The hg index of a researcher is computed as the geometric mean of his h and g indices | Both the h-index and the g-index have positive aspects and drawbacks. This index is supposed to combine the advantages of the two indicators and limit their disadvantages | - Very simple to compute once the h-index and g-index have been obtained - More refined measure - Easy to understand and compare with h-index and g-index - Takes into account the cites of highly cited papers but reduces the impact of very high cited articles | - It is a number with no direct meaning in terms of papers and citations of a scientist - H and g are on different ordinal scales and their equivalence is questionable |
| b-index | [16] | Number of papers of a scientist that belong to the top 10% of papers in that field | - |  | - |
| r-index | [56] | The r-index evaluates the total effective scientific output of a biomedical researcher | The h-index works well in scientific fields where an author’s position on a paper has little significance but works less in other fields (life science) | - It gives a shift in how we evaluated the scientific work and places more value on conduction and leadership as opposed to contribution to the work directed by others | - |
| m-index | [12] | The m-index is defined as the median number of citations received by papers that have a ranking that is equal to or smaller than h | The median is used instead of the average because the distribution of citation counts is usually highly skewed | - The m-index has been found to discriminate better between approved and rejected post doctoral fellowship applicants than the h-index - Allows for comparison of scientists of different age - Can compare scientists in different research domain | - |
| m-quotient | [12; 24; 16; 33] | h-index adjusted for the researcher’s career length | Can be used with the h-index to improve its accuracy | - Insensitive to highly cited work - For young researchers small changes in the h-index can result in large changes in the m-quotient | - |
| Q² index | [12] | The q2 index is the geometric mean of the h-index and the m-index, defined as the square root of the product of the h- and m- indices | This index captures both the number of papers (quantitative dimension) and the impact of the papers (qualitative dimension) in a researcher’s productive (h) core |  |  |
| Crown indicator | [15; 24] | Average number of received citations divided by the aveage numbe that could be expected for publication of the same type published on journals of the same type | By controlling for citation rates for research field, publications year and document type, the crown indicator overcomes limitations of traditional indicators that do not take into account citation practices between different fields | - Allows for comparison of different fields  -controls for differences in citation rates  - correlates well with peer-review judgement  - reflects more accurately than the h-index the lower performance of groups | - it does not take into account the fact that publication from one field is often published in journals from another field  - not readily available  - is only applicable for comparisons of groups of similar sizes |

**3: Indicators of collaboration**

| **Name of the indicator** | **References** | **Definition of indicator** | **Rationale and/or use of this indicator** | **Positive points** | **Negative points** |
| --- | --- | --- | --- | --- | --- |
| Partnership Ability Index (PHI-index) | [52] | The PHI index combines the number of co-authors with the frequency of joint activities between him and his co-authors | - | - This indicator reflects the way an authors is embedded in his collaboration network | None reported |
| Number of co-authored publication | [52; 71; 55; 34] | Number of articles that have been co-authored with one or more organisations | - |  |  |
| Number of articles with international collaboration | [71; 35] | The proportion of publications of an institution that have been co-authored with one or more countries | - |  |  |
| Proportion of long-distance collaborative publication | [71] | The proportion of the publication of a university that have geographical collaboration distance of more than 1000 km | - |  |  |
| d-index (dependence degree) | [10] | Considering a researcher a1 and his co-author a2. the d-index quantifies how much the productivity of all scientific collaboration of a1 differs when a2 is not involved | - |  | The visualisation of the dependence of a researcher is complex. |

4: Indicators of dissemination

| **Name of the indicator** | **References** | **Definition of indicator** | **Rationale and/or use of this indicator** | **Positive points** | **Negative points** |
| --- | --- | --- | --- | --- | --- |
| Citation in medical education books | [45; 38] | Number of articles cited in medical education books | Research aims at the production of new knowledge and new skills than can be used in society | - Can measure indirectly the integration of research into care | No developed or tested |
| Number of presentations at key selected conference | [17] | Number of presentations at key selected conference | Dissemination reflects the extend to which an organisation share their methods and findings with the scientific community |  | - |
| Number of conference held | [52] | - | - |  | - |
| Reporting of research in the news/media | [38; 39; 45; 48] | Number of articles cited in the mass media | Mass media are influential in terms of public opinion and public formation and reporting of research in the news allows patients to be better informed | - Cited sources easy to identify - Media have a significant impact on public debate - Studies show that scientists that interact most with mass media tend to be scientifically productive, have leadership roles and they perceive the interaction to have more positive than negative outcomes | - There are many biases on media reporting of research - Reporting of research might be inaccurate - Reporting of research in the news does not necessarily lead to public policy debate - Clinical research papers are overly cited in newspapers |

**5: Indicators of industrial production**

| **Name of the indicator** | **References** | **Definition of indicator** | **Rationale and/or use of this indicator** | **Positive points** | **Negative points** |
| --- | --- | --- | --- | --- | --- |
| Number of public-private partnerships | [45; 52] | Number of partnerships between an academic research centre and the industry | It is argued that for Translational research organisations (TROs), facilitating external uptake is one objective of translational research. Indeed, TROs may lack the expertise and resources needed to secure approval and commercialise research results or products. Technology transfer or joint development enables TROs to advance technology beyond early-stage while allowing them to avoid high costs. |  | - |
| Number of papers co-authored with the industry | [48] | It is a way of measuring collaboration with industry. Publications resulting from collaboration with university tend to be more applied than basic research |  | - Publications co-authored with the industry are positively associated with researchers productivity in terms of the number of publications and citations although there is no evidence that co-authored publications are more likely to appear in journals with high impact factor than university-only ones. | - It provides incomplete results about collaboration with university |
| Number of spin-off companies created | [48] | Number of spin-off companies created | Technology transfer through spin-out companies has advantages over licensing when the nature of new technology may not be easily patented and when universities seek a greater return on their intellectual property in the long run |  | - In the UK, because of the facility they are created, too many spin-out companies are created, including many of bad quality - Should be measured in terms of success of the companies created, in the long-term and measures should be qualitative rather than quantitative |
| Number of patents | [45; 46; 55; 73; 48; 57] | Number of patents applied for or approved in a definite period of time | Facilitating uptake of research is the ultimate objective of translational research. TROs may lack the expertise and resources needed to secure regulatory approval and commercialise translational breakthrough of their own. A realistic measure of TRO efficacy is a calculation of the number of technologies and treatments that a TRO transfers or jointly develops with third parties. | Patent protection enhances the value of an organisation’s output and attracts future commercial investment | - Does not measure the quality of patents - Many patents come to nothing - Beyond a certain level, higher level of patenting are negatively associated with academic productivity in terms of publications and their ‘basicness’ - Inciting universities to produce more patents can lead to a decrease in the quality of patents (as measured by patent citations) |
| Patent citation count | [25; 67] | Citation of a patent in subsequent patent applications | A highly cited patent is likely to contain technological advances of particular importance that has led to numerous subsequent technological improvements. It is an indicator of the social value or technological impact of a patent | - Good correlation between patent citation and patent count | - 70% of all patents are never cited or cited only once or twice |
| Patent h-index | [25] | Number *h* such that, for a general group of patents, *h* patents received at least h citations from other patents, while other patents received no more than *h* citations | This indicator, like the h-index, combines quantitative and qualitative measures of a patent | - This indicator reflects the importance or impact of patents by balancing quantity and quality | - Lacks sensitivity to performance changes, just like the h-index/it is based on long-term investigations and put newcomers at a disadvantage |
| Citation of research in patents | [6; 17] | Number of patents that cite research produced or funded by an institution by year | This indicator measures the use of research |  |  |

**6: Indicators of health service impact**

| **Name of the indicator** | **References** | **Definition of indicator** | **Rationale and/or use of this indicator** | **Positive points** | **Negative points** |
| --- | --- | --- | --- | --- | --- |
| Citation of research in clinical guidelines | [6; 17; 39; 45 48; 72; 73] | Number of articles that are cited in clinical guidelines | Guidelines facilitate the adoption of research findings and aim to improve quality of healthcare aim change practices | - Measures the way research can impact health - Easier to calculate than the long-term indicator of patients outcomes - Indicator tend to correlate with other indicators of quality such as impact factor | - Long period of time between research result and publication in clinical guidelines - It tends to favour clinical research rather than basic research |
| Citation of research in policy guidelines | [17; 38; 39] | Number of articles that are cited in policy or public health guidelines | It is important to develop ways to measure how research influences patients outcomes | - Measures the impact of research on society | - It takes long time between publication of results until inclusion in policy guidelines |
| Generation of clinical guidelines | [73] | Number of clinical guidelines generated | The ultimate aim of medical research is to improve health and traditional ways of measuring research are not sufficient |  |  |
| Changes in clinical practice | [72] | Actual changes in clinical practices stimulated by a particular scientific finding | The rate of implementation of science into practice is suboptimal. This indicator measures how research is implemented into care | - Measures the real impact of research on care | - very challenging to calculate |
| Patients outcomes | [17; 48; 73] | Measures of improvement of human health | It is argued that the ultimate aim of health research is to improve health so it is supposed to be the most important indicator |  | - It takes long time to be measured - Hard to measure - Difficult to attribute, verify, quantify and meaningfully compare improvements in health |
| Measures of improved health services | [48] | Examples: improved diagnostic and drug response, fulfilling previously unmet clinical needs, reducing waiting time and treatment costs… | Measures the usefulness of medical research |  |  |
| Contribution to reports informing policy makers | [17; 48; 57; 73] |  | University academics generate an evidence base for policy makers and can facilitate the passage of findings into practices. The participation of researchers into public policy committee groups can ease the transition to practice |  |  |
| Public knowledge about a health issue | [17; 48] | Many illnesses can be prevented by changes in public behaviours. Those indicators act as an intermediate outcome indicator of health research | - |  | - Difficulty to attribute to specific research |
| Changes in legislation/regulations | [48; 57] | Changes in legislation or regulation resulting from research results | Research should be used to inform policy makers to make changes positive for the health of the population and therefore should be measured this way. |  | - Difficult to make the link between the publication of research results and the changes in policy - Hard to calculate |
| Clinicians awareness of research | [72] |  | Impact factor alone only measures the awareness of other researchers about research results, not that of clinicians | - It measures the penetration of research into the clinical domain | - Difficult to calculate |

References

1. Adams, J. (2009). The use of bibliometrics to measure research quality in UK higher education institutions. Arch. Immunol. Ther. Exp. (Warsz.) *57*, 19–32.
2. Alonso, S., Cabrerizo, F.J., Herrera-Viedma, E., and Herrera, F. (2010). hg-index: A new index to characterize the scientific output of researchers based on the h- and g-indices. Scientometrics *82*, 391–400.
3. De Araújo, C.G.S., and Sardinha, A. (2011). H-Index of the citing articles: A contribution to the evaluation of scientific production of experienced researchers. Revista Brasileira de Medicina Do Esporte *17*, 358–362.
4. Babineau, M., Fischer, C., Volz, K., and Sanchez, L.D. (2014). Survey of Publications and the H-index of Academic Emergency Medicine Professors. West J Emerg Med *15*, 290–292.
5. Bonastre, J., and de Pouvourville, G. (2006). [How to measure research emerging from hospitals? The case of French comprehensive cancer centres]. Bull Cancer *93*, 1144–1151.
6. Bornmann, L. (2013). What is societal impact of research and how can it be assessed? a literature survey. J Am Soc Inf Sci Tec *64*, 217–233.
7. Bornmann, L., and Daniel, H.-D. (2007). What do we know about the h index? Journal of the American Society for Information Science and Technology *58*, 1381–1385.
8. Bornmann, L., and Daniel, H.-D. (2009). The state of h index research. Is the h index the ideal way to measure research performance? EMBO Rep *10*, 2–6.
9. Bornmann, L., Mutz, R., and Daniel, H.-D. (2009). Do we need the h index and its variants in addition to standard bibliometric measures? Journal of the American Society for Information Science and Technology *60*, 1286–1289.
10. Di Caro, L., Cataldi, M., and Schifanella, C. (2012). The d-index: Discovering dependences among scientific collaborators from their bibliographic data records. Scientometrics *93*, 583–607.
11. Costas, R., and Bordons, M. (2008). Is g-index better than h-index? An exploratory study at the individual level. Scientometrics *77*, 267–288.
12. Derrick, G.E., Haynes, A., Chapman, S., and Hall, W.D. (2011). The Association between Four Citation Metrics and Peer Rankings of Research Influence of Australian Researchers in Six Fields of Public Health. PLoS ONE *6*.
13. Dodson, M.V., de Souza Duarte, M., and dos Santos Dias, L.A. (2012). SP-index: The measure of the scientific production of researchers. Biochemical and Biophysical Research Communications *425*, 701–702.
14. Dorta-González, P., and Dorta-González, M.-I. (2011). Central indexes to the citation distribution: A complement to the h-index. Scientometrics *88*, 729–745.
15. Durieux, V., and Gevenois, P.A. (2010). Bibliometric indicators: quality measurements of scientific publication. Radiology *255*, 342–351.
16. Egghe, L. (2010). The hirsch index and related impact measures.
17. Engel-Cox, J.A., Van Houten, B., Phelps, J., and Rose, S.W. (2008). Conceptual model of comprehensive research metrics for improved Human Health and environment. Environmental Health Perspectives *116*, 583–592.
18. Franceschini, F., and Maisano, D. (2011a). Criticism on the hg-index. Scientometrics *86*, 339–346.
19. Franceschini, F., and Maisano, D. (2011b). Structured evaluation of the scientific output of academic research groups by recent h-based indicators. Journal of Informetrics *5*, 64–74.
20. Franceschini, F., Maisano, D., Perotti, A., and Proto, A. (2010). Analysis of the ch-index: An indicator to evaluate the diffusion of scientific research output by citers. Scientometrics *85*, 203–217.
21. Franceschini, F., Galetto, M., Maisano, D., and Mastrogiacomo, L. (2012). The success-index: An alternative approach to the h-index for evaluating an individual’s research output. Scientometrics *92*, 621–641.
22. Franchignoni, F., Muñoz Lasa, S., Özçkar, L., and Ottonello, M. (2011). Bibliometric indicators: A snapshot of the scientific productivity of leading European PRM researchers. European Journal of Physical and Rehabilitation Medicine *47*, 455–462.
23. Franco, G. (2013). Research evaluation and competition for academic positions in occupational medicine. Archives of Environmental and Occupational Health *68*, 123–127.
24. Froghi, S., Ahmed, K., Finch, A., Fitzpatrick, J.M., Khan, M.S., and Dasgupta, P. (2012). Indicators for research performance evaluation: An overview. BJU International *109*, 321–324.
25. Guan, J.C., and Gao, X. (2009). Exploring the h-lndex at patent level. Journal of the American Society for Information Science and Technology *60*, 35–40.
26. Halbach, O. von B.U. (2011). How to judge a book by its cover? How useful are bibliometric indices for the evaluation of “scientific quality” or “scientific productivity”? Ann. Anat.-Anat. Anz. *193*, 191–196.
27. Hirsch, J.E. (2005). An index to quantify an individual’s scientific research output. Proceedings of the National Academy of Sciences of the United States of America *102*, 16569–16572.
28. Hirsch, J.E. (2007). Does the h index have predictive power? Proceedings of the National Academy of Sciences of the United States of America *104*, 19193–19198.
29. Hofman, V., Gaziello, M.-C., Bonnetaud, C., Ilie, M., Mauro, V., Long, E., Selva, E., Gavric-Tanga, V., Lassalle, S., Butori, C., et al. (2012). [Setting up indicators in biobanking: why and how?]. Ann Pathol *32*, 91–101.
30. Huang, M.-H. (2012). Exploring the h-index at the institutional level: A practical application in world university rankings. Online Information Review *36*, 534–547.
31. Jonkers, K., De Moya Anegon, F., and Aguillo, I.F. (2012). Measuring the usage of e-research infrastructure as an indicator of research activity. Journal of the American Society for Information Science and Technology *63*, 1374–1382.
32. Khan, N., Thompson, C.J., Choudhri, A.F., Boop, F.A., and Klimo, P. (2013a). Part I: The application of the h-index to groups of individuals and departments in academic neurosurgery. World Neurosurg *80*, 759–765.e3.
33. Khan, N.R., Thompson, C.J., Taylor, D.R., Gabrick, K.S., Choudhri, A.F., Boop, F.R., and Klimo, P. (2013b). Part II: Should the h-index be modified? An analysis of the m-quotient, contemporary h-index, authorship value, and impact factor. World Neurosurg *80*, 766–774.
34. Klaić, B. (1999). The use of scientometric parameters for the evaluation of scientific contributions. Coll Antropol *23*, 751–770.
35. Koskinen, J., Isohanni, M., Paajala, H., Jääskeläinen, E., Nieminen, P., Koponen, H., Tienari, P., and Miettunen, J. (2008). How to use bibliometric methods in evaluation of scientific research? An example from Finnish schizophrenia research. Nord J Psychiatry *62*, 136–143.
36. Lee, J., Kraus, K.L., and Couldwell, W.T. (2009). Use of the h index in neurosurgery. Clinical article. J. Neurosurg. *111*, 387–392.
37. Lepori, B., Barré, R., and Filliatreau, G. (2008). New perspectives and challenges for the design and production of S&T indicators. Research Evaluation *17*, 33–44.
38. Lewison, G. (2002). From biomedical research to health improvement. Scientometrics *54*, 179–192.
39. Lewison, G. (2003). Beyond outputs: New measures of biomedical research impact. Aslib Proceedings *55*, 32–42.
40. Luukkonen, T. (1990). Bibliometrics and evaluation of research performance. Ann. Med. *22*, 145–150.
41. Da Luz, M.P., Marques-Portella, C., Mendlowicz, M., Gleiser, S., Freire Coutinho, E.S., and Figueira, I. (2008). Institutional h-index: The performance of a new metric in the evaluation of Brazilian Psychiatric Post-graduation Programs. Scientometrics *77*, 361–368.
42. Martin, B.R. (1996). The use of multiple indicators in the assessment of basic research. Scientometrics *36*, 343–362.
43. Melin, G., and Persson, O. (1996). Studying research collaboration using co-authorships. Scientometrics *36*, 363–377.
44. Moed, H.F. (2009). New developments in the use of citation analysis in research evaluation. Arch. Immunol. Ther. Exp. (Warsz.) *57*, 13–18.
45. Mostert, S.P., Ellenbroek, S.P., Meijer, I., van Ark, G., and Klasen, E.C. (2010). Societal output and use of research performed by health research groups. Health Res Policy Syst *8*, 30.
46. Nicolini, C., and Nozza, F. (2008). Objective assessment of scientific performances world-wide. Scientometrics *76*, 527–541.
47. Opthof, T., and Leydesdorff, L. (2011). A comment to the paper by Waltman et al., Scientometrics, 87, 467-481, 2011. Scientometrics *88*, 1011–1016.
48. Ovseiko, P.V., Oancea, A., and Buchan, A.M. (2012). Assessing research impact in academic clinical medicine: a study using Research Excellence Framework pilot impact indicators. BMC Health Serv Res *12*, 478.
49. Panaretos, J., and Malesios, C. (2009). Assessing scientific research performance and impact with single indices. Scientometrics *81*, 635–670.
50. Patel, V.M., Ashrafian, H., Bornmann, L., Mutz, R., Makanjuola, J., Skapinakis, P., Darzi, A., and Athanasiou, T. (2013). Enhancing the h index for the objective assessment of healthcare researcher performance and impact. J. R. Soc. Med. *106*, 19–29.
51. Pendlebury, D.A. (2009). The use and misuse of journal metrics and other citation indicators. Arch. Immunol. Ther. Exp. *57*, 1–11.
52. Pozen, R., and Kline, H. (2011). Defining Success for Translational Research Organizations. Sci. Transl. Med. *3*, 94cm20.
53. Van Raan, A.F.J. (2006). Comparison of the Hirsch-index with standard bibliometric indicators and with peer judgment for 147 chemistry research groups. Scientometrics *67*, 491–502.
54. Rodríguez-Navarro, A. (2011). A simple index for the high-citation tail of citation distribution to quantify research performance in countries and institutions. PLoS ONE *6*, e20510.
55. Roessner, D., Porter, A.L., Nersessian, N.J., and Carley, S. (2013). Validating indicators of interdisciplinarity: Linking bibliometric measures to studies of engineering research labs. Scientometrics *94*, 439–468.
56. Romanovsky, A.A. (2012). Revised h index for biomedical research. Cell Cycle *11*, 4118–4121.
57. Schapper, C.C., Dwyer, T., Tregear, G.W., Aitken, M., and Clay, M.A. (2012). Research performance evaluation: the experience of an independent medical research institute. Aust Health Rev *36*, 218–223.
58. Schubert, A. (2012). A Hirsch-type index of co-author partnership ability. Scientometrics *91*, 303–308.
59. Schwartz, S., and Lopez Hellin, J. (1996). Measuring the impact of scientific publications. The case of the biomedical sciences. Scientometrics *35*, 119–132.
60. Sharma, B., Boet, S., Grantcharov, T., Shin, E., Barrowman, N.J., and Bould, M.D. (2013). The h-index outperforms other bibliometrics in the assessment of research performance in general surgery: a province-wide study. Surgery *153*, 493–501.
61. Sutter, M., and Kocher, M.G. (2001). Tools for evaluating research output. Are citation-based rankings of economics journals stable? Eval Rev *25*, 555–566.
62. Sypsa, V., and Hatzakis, A. (2009). Assessing the impact of biomedical research in academic institutions of disparate sizes. BMC Med Res Methodol *9*, 33.
63. Todeschini, R. (2011). The j-index: a new bibliometric index and multivariate comparisons between other common indices. Scientometrics *87*, 621–639.
64. Turaga, K.K., and Gamblin, T.C. (2012). Measuring the surgical academic output of an institution: the “institutional” H-index. J Surg Educ *69*, 499–503.
65. Valérie, D., and Pierre, A.G. (2010). Bibliometric idicators: Quality masurements of sientific publication. Radiology *255*, 342–351.
66. De Visscher, A. (2011). What does the g-index really measure? Journal of the American Society for Information Science and Technology *62*, 2290–2293.
67. Wallin, J.A. (2005). Bibliometric methods: Pitfalls and possibilities. Basic Clin. Pharmacol. Toxicol. *97*, 261–275.
68. Waltman, L., and Van Eck, N.J. (2012). The Inconsistency of the h-index. J. Am. Soc. Inf. Sci. Technol. *63*, 406–415.
69. Waltman, L., van Eck, N.J., van Leeuwen, T.N., Visser, M.S., and van Raan, A.F.J. (2011a). Towards a new crown indicator: Some theoretical considerations. Journal of Informetrics *5*, 37–47.
70. Waltman, L., van Eck, N.J., van Leeuwen, T.N., Visser, M.S., and van Raan, A.F.J. (2011b). On the correlation between bibliometric indicators and peer review: reply to Opthof and Leydesdorff. Scientometrics *88*, 1017–1022.
71. Waltman, L., Calero-Medina, C., Kosten, J., Noyons, E.C.M., Tijssen, R.J.W., van Eck, N.J., van Leeuwen, T.N., van Raan, A.F.J., Visser, M.S., and Wouters, P. (2012). The Leiden ranking 2011/2012: Data collection, indicators, and interpretation. J. Am. Soc. Inf. Sci. Technol. *63*, 2419–2432.
72. Weiss, A.P. (2007). Measuring the impact of medical research: moving from outputs to outcomes. Am J Psychiatry *164*, 206–214.
73. Wells, R., and Whitworth, J.A. (2007). Assessing outcomes of health and medical research: do we measure what counts or count what we can measure? Aust New Zealand Health Policy *4*, 14.
74. Wu, Q. (2010). The w-Index: A Measure to Assess Scientific Impact by Focusing on Widely Cited Papers. J. Am. Soc. Inf. Sci. Technol. *61*, 609–614.
75. Zhang, C.-T. (2009). The e-index, complementing the h-index for excess citations. PLoS ONE *4*, e5429.
76. Zhuo, M. (2008). Z factor: a new index for measuring academic research output. Mol Pain *4*, 53.
