# Supplementary material for: Measuring the Outcome of Biomedical Research: A Systematic Literature Review
Source: PLoS One. 2015 Apr 2;10(4):e0122239. doi: 10.1371/journal.pone.0122239 (PMC4383328; doi:10.1371/journal.pone.0122239)
Supplement: S1 Protocol — (DOC) [file pone.0122239.s005.doc]

**A protocol for a systematic review of indicators of cancer research**

1. Background

Cancer, the second cause of mortality in Europe, was accountable for a third of total mortality in 2008 (OECD, 2010). The fight against cancer remains an important challenge, thus the need to accelerate discoveries controlling this disease.

Cancer research faces many challenges: Firstly, the development of new drugs is long (10 to 15 years) and treatments against oncology R&D has the lowest success rate, and by implication the highest cost, of any therapeutic area in the pharmaceutical discovery and development (Kanavos and al, 2009; Littman and al, 2007). When a cancer molecule enters clinical trials, there is only a 5% probability that it will turn out to be a commercially viable product (Kanavos and al, 2009). Secondly, cancer research funding in Europe does not match epidemiological priorities. Research against many cancers (such as colorectal cancer or lung cancer), as measured in terms of publications, is insufficient compared with their epidemiological burden of those cancers, as measured in terms of Disability Adjusted Life Years (DALYs) (Kanavos and al, 2009). Finally, other issues linked to cancer research include: lack of communication and cooperation between clinicians and basic researchers (Butler, 2008), over-regulation (Simon, 2007; Sullivan, 2007: Littman, 2007), fragmentation of infrastructures and lack of qualified investigators (Littman; 2007). Despite strong biomedical research in Europe, fragmentation and duplication of research and the lack of a global vision have been identified as important factors in implementing innovative cancer research and cancer care improvement (EurocanPlus and the IARC, 2008).

Cancer research attracts more attention and funding (Kanavos et al, 2009), hence a need to evaluate it. This study is the first step of a project of development of indicators measuring the outputs of cancer research carried out in research institutes (hospitals, research units…). There are existing research indicators (such as impact factor, citation index, patent citation index…) that have already been developed. The OECD has worked towards the development of science and technology indicators. However those indicators are research inputs rather than outputs (OECD, 2002) and measure the scientific production of countries rather than institutions. It has also been stated that current scientometrics indicators measure research visibility rather than quality (Ugolini, 1997; Zitt and Bassecoulard, 2008). Additional challenges related to the collection of scientometrics indicators: poor quality of data, assessing the position of actors in labyrinth of knowledge flows, and avoiding the mirage of universality that leads to indicators that do not take into account particularities of disciplines of research practices (Zitt and Bassecoulard, 2008).

1. Objectives of the study

The objective of this study is to describe the indicators can be used to measure the production of the research carried out in cancer centres in Europe.

1. Research questions

The research questions are the following:

- What are the existing indicators used to measure research?
- What is their strength and weaknesses?
- In what context are they used?
- Can they be used for assessing the research production from cancer centres

1. Methodology
   1. Search strategy (Literature used, keywords)

The following sources will be used for research:

- Pubmed
- SCOPUS
- Web of Science

The following keywords will be used for research:

| Research outcome* (title/abstract/keyword)  OR  Research output* (title/abstract/keyword)  OR  Bibliometric* (title/abstract/keyword)  OR  Scientific production (title/abstract/keyword)  OR  Scientometric* (title/abstract/keyword) | AND | Indicator* (title/abstract/keyword)  OR  Index* (title/abstract/keyword)  OR  Evaluation (title/abstract/keyword)  OR  Metrics (title/abstract/keyword) |
| --- | --- | --- |

Inclusion criteria:

- Articles that present, discuss or evaluate indicators measuring the scientific production of an institution (hospital, university, laboratory, research unit…), an individual researcher or a country
- Articles in French and English

Exclusion criteria:

- Articles that present or assess indicators measuring only research inputs (such as funding or human resources)
- Articles that use the indicators to measure the scientific production of an institution, country or individual researcher (scientometric or bibliometric studies)
- Articles that present discuss or evaluate indicators measuring scientific production in areas significantly different than health science (eg: physics, computer sciences, humanities…)
- Articles that present discuss or evaluate indicators measuring the quality of a scientific journal
- Articles in languages other than French or English
  1. Assessing the relevance and quality of studies

The quality of studies will be evaluated by the two independent reviewers. As our systematic review does not investigate clinical trials, it is not possible to use many checklists commonly used such as the CONSORT checklist (Wright et al, 2007). The criteria that will be used for selecting the studies are the following:

| Compulsory criteria for selecting articles | Optional criteria for selecting articles |
| --- | --- |
| - The article presents an indicator that is used to measure the scientific production of an institution in terms of outputs - The article clearly explains how the indicator is calculated - The article clearly states for which purposes the indicator is used | - The article evaluates the validity or reliability of the indicator - The article evaluates the feasibility of the indicator - The article evaluates the pertinence of the indicator to measure the scientific production of an institution - The article evaluates the indicator in terms of importance of what is being measured - The article states the possible unintended consequence or perverse effects of measuring the indicator - The article relates the development and implementation of the indicator |

According to the OECD, an indicator is valid when it accurately measures what it is intended to measure. An indicator is reliable when it provides stable results across various populations and circumstances. There are three conditions to assess the feasibility of an indicator: existence of prototypes (whether the measure is in use), availability of internationally-comparable data across countries and cost or burden of measurement (Kelly and Hurst, 2006).

To be selected for our systematic review, articles must meet the three compulsory criteria and at least one optional criterion. Disagreements about the inclusion of a study will be settled by a third person.

We will keep a track of all excluded studies by mentioning the reference and reason for exclusion.

- 1. Data extraction and analysis

Data will be extracted using the form presented in two forms presented in appendix 1 and 2. As it is likely one indicators will be presented or discussed in several articles and that one single article might refer to several indicators, we have decided to use one form to record the articles found (form 1) and one form to record the data about the indicators (form 2).

Then the data will be synthesised and presented using the form presented in appendix 3.

- 1. Reporting of data

The selection of data will be presented using the table presented in Appendix 4, which is a copy of the PRISMA reporting method (Moher et al, 2009).

1. Discussion

We expect to find a high number of research indicators. The challenge of this study will be to determine which indicators can be used for cancer research and to do a pre-selection.

When the systematic review is concluded, we will select the indicators that seem most relevant to assess the outcomes of cancer research according to the scope of the project. Then we will submit this list to a panel of experts concerned by the project and ask them to select the preferred indicators and decide if other indicators should be created.

**References**

EurocanPlus group and the International Agency for Research on Cancer. Feasibility study for coordination of national cancer activities: summary report. *Ecancermedicalscience*. 2008; 2: 84

Faguet GB: *The War on Cancer: An Anatomy of Failure, A Blueprint for the Future*. 2005. New York, NY, Springer, 2006.

Hofman, Véronique, Marie-Clotilde Gaziello, Christelle Bonnetaud, Marius Ilie, Virginie Mauro, Elodie Long, Eric Selva, et al. 2012. « Mise en place d’indicateurs de suivi au sein d’une tumorothèque et/ou d’un centre de ressources biologiques : pourquoi et comment ? » *Annales de Pathologie* 32 (2) (avril): 91-101

Kanavos P, Sullivan R, Lewison G, Schurer W, Eckhouse S, Vlachopioti Z: The role of funding and policies on innovation in cancer drug development. *LSE, ECRM, 2009*

Keating P, Cambrosio A: *Cancer on trial: Oncology as a new style of practice*. University of Chicago Press, 2012

Littman BH, Di Mario L, Plebani M, Marincolas FM: What’s next in translational medicine? *Clinical science* (2007) **112**, 217-227

Moher D, Liberati A, Tetzlaff J, Altman DG; PRISMA Group. Preferred reporting items for systematic reviews and meta-analyses: the PRISMA statement. Int J Surg.2010;8(5):336-41. Epub 2010 Feb 18. Erratum in: Int J Surg. 2010;8(8):658.

OECD (2002), *Frascati manual: Proposed standard practice on surveys on research and experimental development.* OECD Publishing

Kelly E, Hurst J: *Health Care Indicators Project Conceptual Framework Paper*. OECd Publishing, 2006

OECD (2010), *Health at a Glance: Europe 2010,* OECD Publishing. *http://dx.doi.org/10.1787/health_glance-2010-en*

Ugolini D: Assessing research productivity in an oncology research institute: the role of documentation center. *Bull Med Libr Assoc 85(1).* January 1997

Simon R: Lost in translation: Problems and pitfalls in translating laboratory observations to

clinical utility. *Eur J Cancer* 44 (2008) 2707-2713

Sullivan R: Policy challenges for cancer research: a call to arms. *Ecancer* 2007, **1**:53 DOI: 10.3332/ecancer.2008.53

Wright RW, Brand RA, Dunn W, Spindler KP. How to write a systematic review. *Clin Orthop Relat Res*. 2007 Feb;455:23-9

Zitt, M, et E Bassecoulard. « Challenges for scientometric indicators: data demining, knowledge-flow measurements and diversity issues ». *Ethics in Science and Environmental Politics* 8 (juin 3, 2008): 49-60.

Appendix 1: Data extraction form 1

| Full reference of the article |  | | |
| --- | --- | --- | --- |
| Is the article : | | | |
| Presenting the results of surveys to select indicators (such as Delphi survey) | | |  |
| Relating the development of an indicator | | |  |
| A study on the feasibility of an indicator | | |  |
| A study on the validity or reliability of an indicator | | |  |
| An evaluation of the impact of developing or measuring an indicator | | |  |
| Any other form of evaluation of an indicator | | |  |
| Number of indicators presented in this article: | | |  |
| Name of this (or those) indicator(s): | |  | |

Appendix 2: Data extraction form 2

| Name of the indicator |  | |
| --- | --- | --- |
|  | How the indicator is calculated |  |
| Reference of the article |  | |
|  | Year |  |
| How the indicator is used: | In what context: |  |
|  | Who uses the indicator |  |
| Positive points of the indicator |  | |
| Negative points of the indicator |  | |
|  | Unintended effects or perverse consequences of measuring the indicator: |  |
| Impact of measuring the indicator: |  | |
| Comments: |  | |

Appendix 3: Data presentation form

| Reference of the article | Indicator presented or discussed | How indicator is calculated | How indicator is used | Positive points of indicator | Negative points of indicator |
| --- | --- | --- | --- | --- | --- |
|  |  |  |  |  |  |
|  |  |  |  |  |  |
|  |  |  |  |  |  |

Appendix 4: Studies selection presentation form

**Screening**

**Included**

**Eligibility**

**Identification**

Records identified through database searching
(n = )

Additional records identified through other sources
(n = )

Records after duplicates removed
(n = )

Records screened
(n = )

Records excluded
(n = )

Full-text articles assessed for eligibility
(n = )

Full-text articles excluded, with reasons
(n = )

Studies included in qualitative synthesis
(n = )
